# Marine Natural and Nature-Inspired Compounds Targeting Peroxisome Proliferator Activated Receptors (PPARs)

**DOI:** 10.3390/md21020089

**Published:** 2023-01-26

**Authors:** Enrico D’Aniello, Pietro Amodeo, Rosa Maria Vitale

**Affiliations:** 1 Department of Biology and Evolution of Marine Organisms, Stazione Zoologica Anton Dohrn, Villa Comunale, 80121 Naples, Italy; 2 Institute of Biomolecular Chemistry, National Research Council (ICB-CNR), Via Campi Flegrei 34, 80078 Pozzuoli (NA), Italy

**Keywords:** PPARs, drug discovery, marine natural products, PPAR modulators

## Abstract

Peroxisome proliferator-activated receptors α, γ and β/δ (PPARα, PPARγ, and PPARβ/δ) are a family of ligand-activated transcriptional factors belonging to the superfamily of nuclear receptors regulating the expression of genes involved in lipid and carbohydrate metabolism, energy homeostasis, inflammation, and the immune response. For this reason, they represent attractive targets for the treatment of a variety of metabolic diseases and, more recently, for neurodegenerative disorders due to their emerging neuroprotective effects. The degree of activation, from partial to full, along with the selectivity toward the different isoforms, greatly affect the therapeutic efficacy and the safety profile of PPAR agonists. Thus, there is a high interest toward novel scaffolds with proper combinations of activity and selectivity. This review intends to provide an overview of the discovery, optimization, and structure–activity relationship studies on PPAR modulators from marine sources, along with the structural and computational studies that led to their identification and/or elucidation, and rationalization of their mechanisms of action.

## 1. Introduction

Peroxisome proliferator-activated receptors (PPARs), a family of ligand-dependent transcription factors, belong to the superfamily of nuclear receptors, which regulate the expression of genes involved in a variety of biological processes mainly related but not limited to lipid and glucose metabolism [1]. The three subtypes identified so far, namely PPARα, PPARγ (existing in two isoforms: PPARγ1 and PPARγ2), and PPARβ/δ, are characterized by different functions and expression levels in tissues and organs [2]. PPARα plays a key role in lipid catabolism and is highly expressed in organs and tissues with high lipid oxidation rates such as liver, brown adipose tissue, heart, intestinal tract, and kidney. PPARγ regulates glucose homeostasis, storage of lipids, and adipocyte differentiation, and is mainly expressed in the adipose tissue and in the immune system [1]. The third isoform, PPARβ/δ, is ubiquitously expressed and, albeit its role has not yet been fully elucidated, it enhances fatty acid oxidation in skeletal muscle and ameliorates obesity and insulin resistance in obese animals [3]. PPARs are considered sensors for a wide range of lipid molecules, including endogenous and exogenous fatty acids and their metabolized products from microbiota [4,5] or intracellular signaling pathways [6], endo- and phyto-cannabinoid-related molecules [7,8,9], and other classes of natural and synthetic compounds. In this view, they are well-recognized targets for the treatment of hyperlipidemia [10] and type-2 diabetes [11]. Besides their role in energy homeostasis, PPARs have also exhibited anti-inflammatory [12] and neuroprotective effects in several studies using in vivo models of neurodegenerative disorders [13,14,15], including Alzheimer’s disease, Parkinson’s disease, traumatic brain injury, and retinopathy [16], thus further expanding the therapeutic application of PPAR agonists beyond the treatment of metabolic-related diseases. Fibrates and thiazolidinediones represent the first classes of synthetic PPARα and PPARγ agonists on the market. However, albeit fibrates are generally well-tolerated, adverse effects such as myopathy and rhabdomyolysis [17] and acute kidney injury [18] have been reported. Meanwhile, the clinical use of thiazolidinediones has been impaired by severe side effects including weight gain, fluid retention, and bone fractures [19], calling for a next-generation of PPAR agonists characterized by a higher safety profile. Since these adverse effects could arise from an unbalanced activation of the different isoforms, an attractive strategy could be the use of dual or pan-agonists able to simultaneously target two or more PPAR isoforms, or partial agonists, which exert a selective/reduced transcriptional activity with respect to the full agonists [20,21,22]. In this context, the elucidation of the molecular mechanisms responsible for proper binding and the activation of this class of receptors represents a critical initial step for the selection, identification, and design of novel future PPAR agonists endowed with a desired activity profile. 

## 2. Domain Organization of PPARs and Their Mechanism of Action

**Figure 1:**
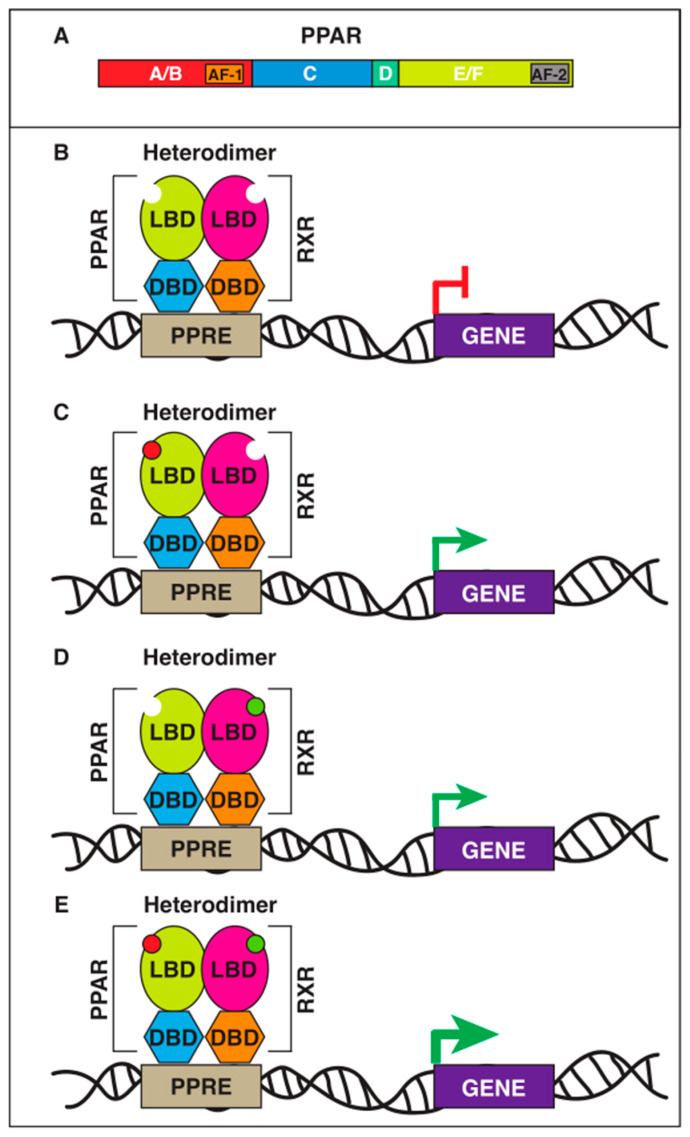
Schematic representation of PPAR domain organization and PPAR-RXR heterodimer activation. (**A**) A/B domain (N-terminal variable domain with transactivating AF-1 domain), C (DBD, which contains two Zinc finger (Znf) motifs), D domain (a linker domain), E/F domain (LBD plus transactivating AF-2 domain). (**B**–**E**) Schematic representation of PPAR/RXR permissive heterodimers and different ways it can be activated by ligands of either PPAR (**C**), RXR (**D**) or by both resulting in a synergistic activation (**E**) as shown by the increased size of the green arrow. Red circles represent PPAR ligands, green circles RXR ligands.

Like other nuclear receptors, the multidomain organization of PPARs consists of four domains harboring specific functions (Figure 1): N-terminal–A/B domain, containing the ligand-independent activation function 1 (AF-1); C domain, containing the DNA binding domain (DBD); D domain, a hinge region; C-terminal E/F domain, featuring the ligand-binding domain (LBD), including the ligand-dependent activation function 2 (AF2) [12]. PPARs act as obligate heterodimers with retinoid X receptors (RXR α, β and γ), another family of nuclear receptors. They form a heterodimeric transcriptional complex which recognizes specific DNA sequence elements, termed peroxisome proliferator response elements (PPREs), upstream of their target genes. Such heterodimers are defined “permissive”, because they can be activated by either PPAR or RXR ligands, and the simultaneous occupancy of both sites gives rise to a synergistic response (Figure 1) [23,24]. The activity of PPAR/RXR heterodimers is finely regulated by their association with coactivators or corepressors. In the presence of corepressors, such as silencing mediator of retinoic acid and thyroid hormone receptor (SMRT) and nuclear receptor corepressor (NcoR), the transcriptional activity is inhibited [25]. Upon binding, ligands trigger a conformational change in the LBD, which induces the dissociation of the corepressors and the recruitment of coactivators, such as the p160 family, p300/CBP, and PGC-1, leading to the transcription of the target genes [26]. Besides the activation and repression of the transcriptional process, there is a third mechanism called “transrepression”, i.e., the ligand-dependent ability to interfere with the activities of other transcription factors mediating the inflammatory response, such as members of the nuclear factor-κB (NF-κB) and activator protein-1 (AP-1) families. This mechanism is believed to underpin the anti-inflammatory effect of PPARs [27].

### PPAR-Ligand Binding Domain (LBD) 

**Figure 2:**
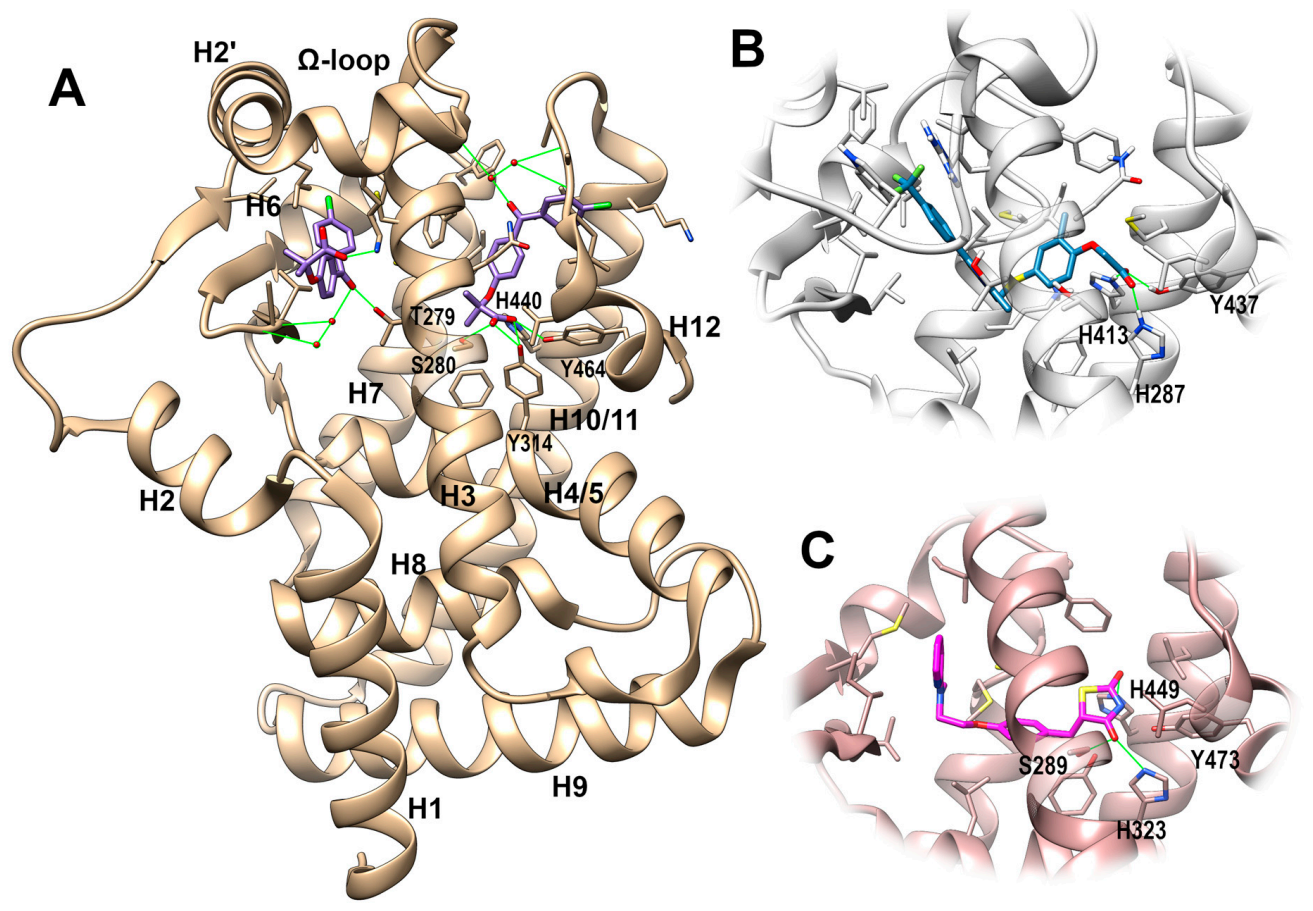
Representative *X*-ray complexes of PPAR LBDs. (**A**): Full view of PPARα LBD (tan) with the full agonist fenofibrate (purple sticks), PDB id: 6LX4. (**B**): Detail of the ligand binding site of PPARδ LBD (gray) with the full agonist GW501516 (steel blue sticks), PDB id: 5U46. (**C**): Detail of the ligand binding site of PPARγ LBD (rosy brown) with the full agonist rosiglitazone (magenta sticks), PDB id: 5YCP. Protein sidechains within 4 Å from the ligand are shown in stick representation. Oxygen, nitrogen, and sulfur atoms are colored in red, blue, and yellow, respectively. Direct and water-mediated H-bonds engaged by the ligands are shown as green lines.

The availability of an ever-increasing number of crystallographic structures of PPAR-LBDs, either in apo forms, or in complex with ligands endowed with different molecular scaffolds, activities and potencies, has greatly contributed to the elucidation of the molecular determinants responsible for the binding and activation of this class of receptors. LBD is characterized by a roughly globular structure of ~270 residues, formed by 13 α-helices, named H1-H12 and H2’, and a four-strand β-sheet region [28] (Figure 2), with a large three-branched (I–III) binding site, described in the literature as being Y- or T-shaped to accommodate the ligand. Branch I, located near helix H12, contributes to the site along with helices H3, H5, H11, and represents the anchoring point for the polar head of the ligands. The external faces of helices H3, H5, and H12 form the AF2 surface, which represents the binding region of the LXXL motif of coactivators. Branch II instead is close to the β-sheet and comprises helices H2’, H3, H6, and H7. It consists of a mainly hydrophobic region hosting the lipid tail of the ligands, while branch III, including both hydrophobic and hydrophilic regions, spans the β-sheet and helices H2, H3, and H5. Besides the orthosteric ligand binding site, recent structural studies have shown that PPAR ligands can also bind a so-called alternate site or X arm, which comprises regions partially overlapping branch II and the flexible Ω-loop connecting helices H2’ and H3 [29,30]. Canonical full agonists engage in H-bonds with a tyrosine residue (473 in PPARγ and 464 in PPARα) located on helix H12, and induce the correct repositioning of this helix to form the co-activator binding surface. Antagonists, instead, can destabilize the H12 helix through their bulkier substituents, thus altering the AF2 surface [28]. However, PPAR’s mechanism of activation cannot be fully explained by the simple “on-off” switch of helix H12. Indeed, the activity of PPAR partial agonists does not necessarily correlate with the stabilization of the H12 helix. An NMR study using hydrogen–deuterium (H/D) exchange [31] shows, in fact, that partial agonists preferentially interact with other regions of the LBD, in particular the β-sheet. NMR studies also provided new insights into the dynamic mechanism governing PPAR activation, suggesting that the LBDs exist as an ensemble of multiple conformations which are differently stabilized upon ligand binding [32]. This decoupling between receptor activation and binding to helix H12 is also observed in the binding/activation profiles exhibited by different fatty acids. Although the binding modes to PPARα-LBD of compounds such as oleic acid, arachidonic acid, and EPA involve interactions with helix H12, they do not trigger any receptor activation. Other fatty acids instead, such as palmitic and stearic acids, both bind helix H12, and activate the LBD [30]. The antidiabetic efficacy of partial agonists that bind at branch II and/or the alternate site can be explained by the inhibition of the Cdk5 (cyclin-dependent kinase 5)-mediated phosphorylation of a serine (Ser273 in the PPARγ isoform 1 or Ser 245 in the isoform 2) [33]. Recently, the co-binding of synthetic ligands at the orthosteric pocket and either dietary/bacterial medium-chain, or endogenous long-chain unsaturated fatty acids at an alternate binding site, has been observed [29]. The co-binding affects the conformation of the AF-2 surface and synergistically enhances coactivator interaction [29]. The simultaneous binding of two ligands at two sites has been also observed in *X*-ray structures of PPARα in complex with fibrates [30] (Figure 2), adding a further layer of complexity to the activation mechanism of this family of nuclear receptors.

## 3. Marine Natural and Nature-Inspired Compounds as PPAR Modulators 

Natural compounds from marine organisms are gaining ever increasing attention in drug discovery due to the high diversity of their chemical scaffolds, albeit their exploitation as PPAR modulators has only been superficially scratched. To date, the main source of marine PPAR ligands are algae, sponges, and fungi (Table 1). 

The identification of these ligands still mainly relies on the bio-assay guided screening of marine extracts or small libraries of purified compounds, since only in few cases the process has been driven by computational approaches. However, molecular docking techniques are considerably assisting the synthesis of new compounds inspired by the ever-growing ensembles of novel molecular scaffolds discovered in the screenings. These combined computational and synthetic efforts are strongly helping in unveiling the role of the relevant ligand structural features in PPAR modulation and/or selectivity toward the different isoforms. In addition, the ongoing determination of experimental structures of the ligand binding domain (LBD) of the three PPAR isoforms in complex with either full or partial agonists is indeed contributing to clarifying the mechanisms underlying the multiple ligand binding and activation modes of PPARs to better characterize the modulation of the transcriptional cascade responsible for the proper regulation of their target genes.

According to their chemical scaffold and/or functional groups, the compounds can be grouped into the following structural classes: (1) oxygenated/unsaturated fatty acids free (FFAs), (2) mero/nor-terpenoids, (3) lactones, (4) phtalides, (5) phosphonates, and (6) miscellaneous.

### 3.1. Oxygenated/Unsaturated FFAs 

**Figure 3:**
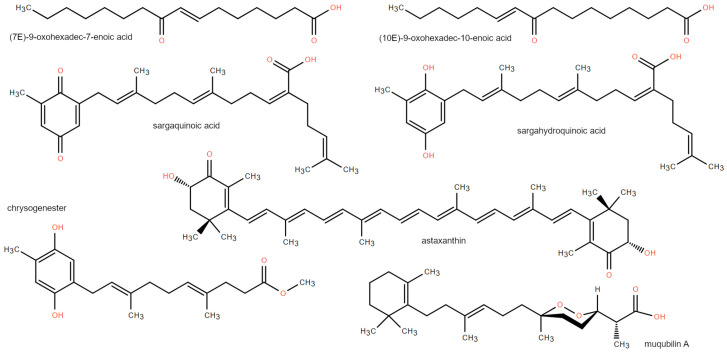
2D structures of oxygenated/unsaturated fatty acids and mero/nor-terpenoids discussed in Section 3.1 and Section 3.2.

Some hydroxylated derivatives of polyunsaturated fatty acids (PUFAs), such as 12-HETE and 13-HODE, well-known PPAR ligands, have been found in the red alga *Chondrus crispus*, where they are likely involved in defense mechanisms [49]. Omega-3-fatty acids, abundant in fish oil, have been reported to alleviate several chronic inflammatory conditions. Since these fatty acids are highly susceptible to oxidation, it is reasonable to hypothesize that their oxidized form mediates the anti-inflammatory effects of fish oil. Indeed, the oxidized eicosapentaenoic acid (EPA), but not the native EPA, potently activates PPARα [51], thus down-regulating the expression of leukocyte adhesion receptor and ultimately exerting an inhibitory activity on leukocyte interactions with the endothelium, as found in both in vitro, and in vivo inflammation models. The brown alga *Lessonia spicata* accumulates unsaturated FFAs such as linoleic (C18:2), linolenic (C18:3), and arachidonic (C20:4) acids, direct precursors of oxylipins, using them as a defense against grazing [50]. These PUFAs are ligands of all the three PPAR isoforms in the micromolar range [6,52]. Moreover, linoleate and arachidonate can be oxidated to 13-HODE and 15-HETE [53], respectively, globally functioning as dual PPARα/γ agonists, since 15-HETE is a PPARγ ligand, while 13-HODE is a dual PPARα/γ agonist [53,54]. Other PPARα/γ dual agonists have been identified with a bioassay-guided screening of organic extracts from the Norwegian Biobank of Arctic Marine Organisms. Specifically, they are two isomeric oxo-fatty acids from the microalgae *Chaetoceros karianus*, namely (*7E*)-9-oxohexadec-7-enoic acid and (*10E*)-9-oxohexadec-10-enoic acid (Figure 3), also showing an opposite potency toward the two isoforms, albeit at high concentration (100 μM) [44]. In this view, these two compounds have been used as a starting point for the design and synthesis of a selective PPARα agonist (EC_50_ 47 μM) by replacing the α,β-unsaturated ketone moiety with a bioisosteric isoxazole ring [55]. Molecular modeling studies have shown that this compound engages a network of H-bonds in the PPARα ligand binding domain (LBD) between its carboxylate moiety and PPARα polar amino acids, including Ser280, Tyr314, His440, and Tyr464, which recapitulate the canonical binding mode of full PPAR agonists.

### 3.2. Mero/Nor-Terpenoids

The organic extract from the brown alga *Sargassum yezoense* resulted as the most potent activator of PPARα/γ transcriptional activity in a bioassay-guided screening of marine product extracts [47]. Such an extract increased the adipocyte differentiation in 3T3-L1 cells by 5.9-fold, in a manner comparable to the effect of troglitazone (7.3-fold), by inducing the expression of adipogenic genes. The subsequent purification of the active ingredients responsible for PPARα/γ activation led to the identification of two major plastoquinone-type meroditerpenoids, namely sargaquinoic acid (SQA) and sargahydroquinoic acid (SHQA), shown in Figure 3. SQA shows similar potency toward both PPARα and PPARγ activation (2.6-fold and 3.0-fold increases, respectively), while the effect of SHQA is higher (4.3-fold) on PPARα than PPARγ (2.1-fold). SQA has little effect on PPARδ activation (below 2.0-fold), and no concentration-dependency is observed. Direct binding to PPARγ of SQA and SQHA was assessed by a fluorescent binding assay, with an IC_50_ of 255 and 725 nM, respectively; while, the IC_50_ of troglitazone, used as the positive control, is 658 nM. When assayed as single compounds, SQA and SHQA significantly enhanced adipocyte differentiation in 3T3-L1 and increased lipid accumulation of adipocytes in a comparable manner to troglitazone, as well as the triglyceride content up to 5.6- and 6.3-fold, respectively, in differentiated adipocytes [47]. The meroterpene chrysogenester isolated from the jellyfish-derived fungus *Penicillium chrysogenum* J08NF-4, and shown in Figure 3, was found to activate PPARγ in a concentration-response manner, showing at 20 μM a comparable potency to rosiglitazone at 5 μM. Modeling studies suggested that it binds to LBD of PPARγ in the same orientation adopted by amorfrutin, a natural compound whose complex with PPARγ has been solved by *X*-ray crystallography (PDB id: 2YFE). Chrysogenester interacts with LBD through H-bond interactions with Ser342 of the β-sheet, and with Cys285 and Arg288 of the helix H3. A further in vitro characterization of chrysogenester showed that it is able to suppress PPARγ-mediated inflammatory responses, leading to the down-regulation of the NF-κB signaling pathway and, subsequently, of proinflammatory mediators [37]. Using a luciferase-based assay screening, the carotenoid astaxanthin (AX) (Figure 3) was identified for its PPAR activity among 900 Korean natural extracts and compounds derived from plant and marine organisms. AX occurs in a wide variety of pink-to-red colored marine organisms, including microalgae, salmon, and crustaceans. Luciferase reporter gene assay shows that AX activates PPARα at 10, 50, and 100 μM, producing 1.98, 2.02, and 2.03-fold increases, respectively. It also acts as antagonist at PPARγ whilst having no activity on PPARβ/δ [56]. Due to its activity as a PPARα agonist, AX at 10 μM has been demonstrated to induce hypolipidemic effects in hepatocytes, being able to reduce the cellular cholesterol and TG concentrations by −14 and −20%, respectively, in HepG2 cells. The hypolipidemic effects of AX in vivo have been reported by several groups: oral administration of AX (50 mg/kg/day) for 22 weeks reduces non-esterified fatty acid and TG concentrations, and increases high-density lipoprotein cholesterol levels in an animal model [57] and in humans [58]. Moreover, AX supplementation in Wistar rats with alloxan-induced diabetes for 23 days significantly reduces plasma TG levels [43]. Using a virtual-screening approach to identify putative PPAR/RXR multiligands in the in-house developed Structurally Oriented Molecular DataBase (StOrMoDB) containing approximatively 350 compounds of marine origin, we identified the norterpene cyclic peroxide (−)-muqubilin A from a marine sponge *Diacarnus erythraeanus* as the best hit (Figure 3). Molecular docking and molecular dynamics simulations confirmed its propensity to effectively bind the abovementioned targets. The computational results revealed that this compound is able to bind the LBD of PPARα and PPARγ as a canonical full agonist. This prediction was confirmed by luciferase assay, where we found that muqubilin strongly activates both isoforms in a low micromolar range (1–10 μM), and in a concentration-dependent manner [46]. 

### 3.3. Polyketides and Lactones

**Figure 4:**
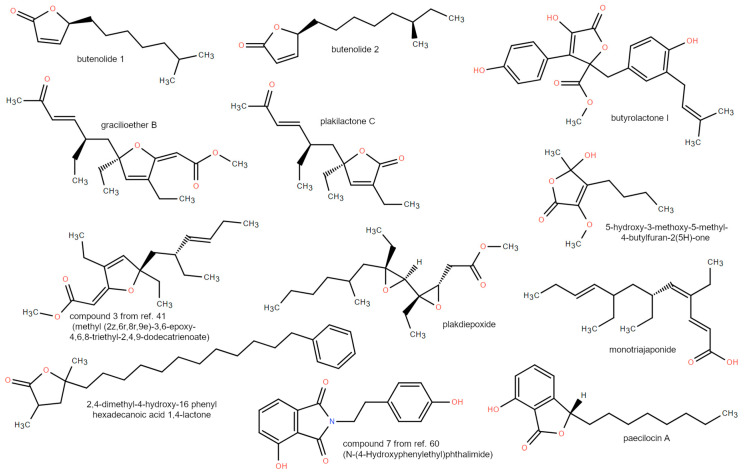
2D structures of polyketides, lactones, and phtalides discussed in Section 3.3 and Section 3.4.

Two butenolides (Figure 4) bearing an alkyl side chain from a marine-derived *Streptomyces* strain were identified using reporter gene assay screening for novel scaffolds of PPAR agonists. Both compounds induce activation of PPARα and PPARγ in a 1–10 μM range and in a concentration-dependent manner, with a specificity of ca 3-4-fold toward PPARα [34].

**Figure 5:**
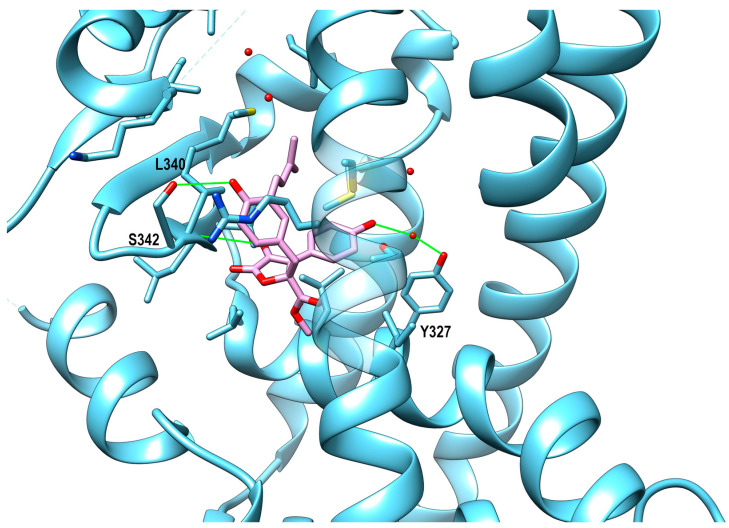
*X*-ray structure of PPARγ LBD (colored in light-blue) with the partial agonist butyrolactone I (represented in stick and colored in pink), PDB id: 6L89 (chain B). Protein sidechains within 4 Å from the ligand are shown in stick representation. Oxygen, nitrogen, and sulfur atoms are colored in red, blue, and yellow, respectively. Direct and water-mediated H-bonds engaged by the ligands are shown in wire green.

During a phenotypic screening of secondary metabolites derived from the marine fungus *Aspergillus terreus*, butyrolactone I (Figure 4), a known CDK5 inhibitor, was discovered as a potent enhancing compound of adiponectin production in the adipogenesis model of human bone marrow-derived mesenchymal stem cells (hBM-MSCs). Generally, inhibitors of CDK5 down-regulate adiponectin production by counteracting the phosphorylation of PPARγ from the protein kinase. However, the potent effect of butyrolactone I does not correlate with its weak activity as a CDK5 inhibitor alone. Therefore, an accurate target identification among various nuclear receptors involved in adiponectin biosynthesis (PPARα, PPARγ, PPARδ, GR, ER, and LXR) was performed for butyrolactone I by using time-resolved fluorescence resonance energy transfer (TR-FRET)-based nuclear receptor binding assays. It revealed that butyrolactone I is able to significantly bind only PPARγ (K_i_ 2.64 µM). Butyrolactone I also significantly increases PPARγ transactivation, behaving as a PPARγ partial agonist, as confirmed by the crystal structure of the complex where the compound does not directly interact with Tyr473 on H12, the major interactive motif of PPARγ full agonists. This ligand occupies a subpocket comprising helices H3 and H5, and the β-sheet of the PPARγ LBD, where it is stabilized by hydrophobic interactions and H-bonds involving the Ser342 sidechain, the backbone oxygen atom of Leu340, and the sidechain of Tyr327 [36] (Figure 5). The biochemical investigation of a library containing 13 oxygenated polyketides extracted from the sponge collected at the Fiji Islands, *Plakinastrella mamillaris*, led to the discovery of gracilioether B and plakilactone C as selective and covalent PPARγ ligands (Figure 4). Transactivation assays of these compounds revealed an EC_50_ of ≈5 and 2 μM. Both compounds bind the PPARγ LBD by a Michael addition reaction involving Cys285 and their α,β-unsaturated ketone moiety. The resulting positive activation regulates the expression of PPARγ downstream target genes in both HepG2 cells and macrophages. In this study, the authors also identified other non-covalent ligands, namely graciliother C (EC_50_ of 10 μM) and methyl esters **1** and **2** in [38], with the latter two acting as PPARγ antagonists whose binding modes were characterized by molecular docking and dynamics studies. All the docked compounds lie between helix H3 and the β-sheet of PPARγ LBD, in close proximity of Cys285 and Arg288. However, the methyl esters 1 and 2 adopt a different orientation in comparison to the non-covalent agonist graciliother C, showing a loss of stabilizing polar interactions, potentially responsible for their inability to activate the receptor [38]. The marine-derived furanone, 5-hydroxy-3-methoxy-5-methyl-4-butylfuran-2(*5H*)-one (Figure 4), isolated from the fermentation broth of the fungus *Setosphaeria sp SCSIO41009*, upregulates PPARα with a lipid-lowering effect, albeit the exact mechanisms were not fully understood since the simultaneous administration of PPARα antagonists can only partially inhibit such effects [35]. The compound 2,4-dimethyl-4-hydroxy-16 phenyl hexadecanoic acid 1,4-lactone (see Figure 4), first isolated from the deep-water sponge *Plakortis nigra*, was identified as a PPAR agonist during a high-throughput screening of marine natural product libraries, and its activity profile was investigated after the stereo-controlled synthesis of its four stereoisomers [48]. The results reported showed that all the stereoisomers behave as dual PPARα/δ agonists in a low micromolar range when tested on mouse receptors, with the most active compound having an EC_50_ of 12 μM for PPARα, 9 μM for PPARδ, and >100 μM for PPARγ. Among the polyketides isolated from the Chinese sponge *Plakortis simplex*, active on PPARs at the luciferase assay (Figure 4), three furanylidene acetates (only the most active of the three acetates, compound **3** in [41], is shown in Figure 4) and plakdiepoxide have been identified as selective PPARγ agonists, while monotriajaponide was found to act as a dual PPARα/γ agonist [41].

### 3.4. Phtalides

A series of naturally occurring phthalide derivatives, namely paecilocins A–C, isolated from the jellyfish-derived fungus *Paecilomyces variotii*, were screened using the LanthaScreenTM TR-FRET PPAR-γ competitive binding assay. These analyses revealed that the affinity of paecilocin A, shown in Figure 4, is comparable to that of rosiglitazone at 100 μM [39]. On the basis of these results, paecilocin A, as racemate and its analogs, were synthetized to identify the structure–activity relationships of this scaffold. This study showed that the occurrence of the hydroxyl group on the benzene ring is critical, since the binding was reduced when it was blocked by a benzyl or methyl group. Moreover, the substitution of a bulky group at the stereocenter was detrimental to the binding. The racemate (compound **7** in [39]) displayed higher binding affinity than the natural compounds, suggesting that the *R* enantiomer performs better than the naturally-occurring *S* enantiomer. The binding modes of the two enantiomers along with compound 6, which differs from compound **7** in the position of the hydroxyl group on the lactone ring, were evaluated by molecular docking and luciferase assay. In docking studies, **7R** ranked over **7S**, and both performed better than both enantiomers of compound **6** [39]. Compound **7R** forms H-bonds with Tyr473, His449, His343, Ser289, and Glu286 in the PPAR-γ LBD. Albeit at binding assay, compound **6** and **7** are comparable with rosiglitazone, and in vitro analyses with luciferase assays showed that they are weaker agonists than rosiglitazone, and that only compound **7** shows a concentration–response increase in a 0.1–10 μM range [39]. Starting from these results, a series of N-substituted phthalimide derivatives were synthesized producing very interesting results. Specifically, the most active compound within the series, named compound **7** in [59] (Figure 4), shows a significant concentration-dependent PPAR-γ activation in a luciferase assay using rat liver Ac2F cells, with an EC_50_ of 0.67 μM, and a potency comparable to that of rosiglitazone at 10 μM. Docking studies on this synthetized compound suggested that it forms H-bonds with Tyr473, His323, and Ser289 in the PPAR-γ binding pocket [59]. Furthermore when analyzed both in vitro and in vivo, it exerted anti-inflammatory activity by suppressing the induction of pro-inflammatory factors, including inducible nitric oxide synthase (iNOS), nitric oxide (NO), cyclooxygenase 2 (COX-2), tumor necrosis factor (TNF-α), interleukin-1(IL-1), and interleukin-6 (IL-6). In addition, it enhanced the expression of anti-inflammatory factors, such as arginase-1 and interleukin-10 (IL-10). In a rat paw edema model, intraperitoneal injection of PD1 (corresponding to compound **7** in [59]) significantly reduced paw swelling [60].

### 3.5. Phosphonates

**Figure 6:**
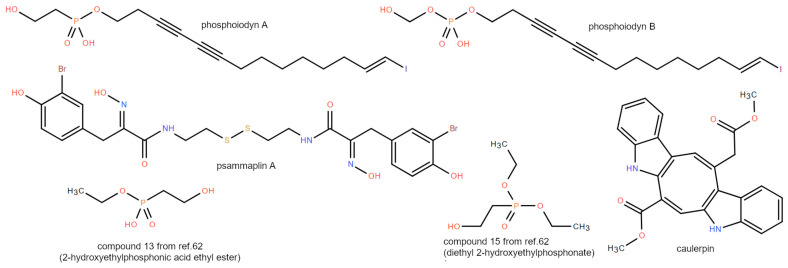
2D structures of phosphonates and miscellaneous compounds discussed in Section 3.4 and Section 3.5.

Two phosphorus-containing iodinated polyacetylenes isolated from a Korean marine sponge *Placospongia sp.*, phosphoiodyns A and B shown in Figure 6, only differing from each other in the occurrence of a C-1’-P bond in compound A, were tested for human PPARs using a bioactivity-guided fractionation. Phosphoiodyn-A shows >200-fold greater selectivity toward PPARδ than PPARα and PPARγ, with an EC_50_ of 23.7 nM; on the contrary, phosphoiodyne B exhibits no PPARδ activity at a concentration of 10 μM, suggestive of a critical role of the C–P bond in PPARδ agonism [42]. The phosphonate core was further investigated for the design and synthesis of novel human PPARδ agonists. Two simplified phosphonate esters, compounds **13** and **15** in [61], display potent activity with an EC_50_ of 0.078 μM and 0.112 μM, respectively. The highly-charged phosphonic is instead inactive toward all PPAR isoforms, probably due to its high hydrophilicity hindering its membrane permeability. The phosphonate esters **13** and **15** also exhibit significant neuroprotective activity, suggesting that these compounds could be useful to trigger proliferation or neurogenesis [61].

### 3.6. Miscellaneous

The bioassay-guided fractionation and isolation of an active extract from the marine sponge *Pseudoceratina rhax* led to the identification and the characterization of psammaplin A (Figure 6) as s PPARγ agonist (EC_50_ 5.8 μM) in an MCF-7 cell-based reporter assay. Docking studies suggested that this compound forms hydrophobic interactions with Leu255, Leu270, Ile281, Gly284, Val339, Met340, and Ile341 and H-bonds with Glu259, Gln286, and Ser342 within the PPARγ LBD. Psammaplin A also induces differentiation and subsequent apoptosis in human breast tumor cells in vitro [40]. The bisindole alkaloid caulerpin (Figure 6), from the green alga *Caulerpa cylindracea*, was recently identified as a dual PPARα/γ agonist using a computational approach, a result validated by in vivo, ex vivo and in vitro assays. Caulerpin engages mainly in hydrophobic interactions within the LBD of both PPAR isoforms in a sub-pocket far from helix H12, which is typically stabilized by canonical full agonists. Instead, the binding mode of caulerpin strongly resembles that adopted by magnolol in the *X*-ray complex with PPARγ [62]. These results are in agreement with the luciferase assay, showing a bell-shape curve in the 1–30 μM range, suggestive of partial agonist activity for this compound for PPARα/γ. Due to its effects on metabolism, caulerpin has been proposed as a causal factor for the metabolic and behavioral disorders observed in *Diplodus sargus*, which feeds on *C. cylindracea* [45].

## 4. Conclusions

To date, the search for novel PPAR modulators has only marginally exploited the structural diversity of molecular scaffolds from marine natural products. However, although these studies are still in their infancy, interesting results have already been obtained in terms of structure–activity relationships derived from small libraries of natural and nature-inspired compounds. For instance, these preliminary analyses have allowed for the identification of new lead compounds functioning as covalent PPARγ agonists, gracilioether B and plakilactone, the lead-to-hit optimization of N-substituted phthalimides, or the discovery of the different activities toward PPARδ due to the oxygen–carbon replacement of phosphoiodyns A and B. Moreover, as in the case of the Mediterranean invasive algae *C. cylindracea*, the discovery of metabolites active on PPARs paves the way for the exploitation of biomasses from marine invasive species for nutraceutical/pharmacological applications. 

At a methodological level, the relevance of structural and computational studies in the identification of the structural determinants responsible for binding and activation of these nuclear receptors clearly shows the strategic power of multidisciplinary approaches. Their contribution is particularly relevant in the characterization and optimization of novel PPAR modulators with graded profiles in potency and selectivity, from the complex and variate libraries of natural compounds typical of many classes of marine species. Finally, in addition to the search for novel scaffolds, the identification of known PPAR agonists, mainly from fatty acid metabolism, such as those found in edible algae and crustaceans, provides useful information and potential contributions to the valorization of marine resources. The occurrence of PPAR agonists in seafoods, due to their beneficial effects on a wide range of metabolic, inflammatory, and neurodegenerative disorders, is indeed expected to greatly contribute to their nutritional value. 

## Figures and Tables

**Table 1:** Natural compounds targeting PPARs discussed in the review and the corresponding marine sources.

PPAR Isotypes	Compounds	Marine Source
PPARα	butenolides	marine-derived *Streptomyces* strain [34]
PPARα	furanone	fungus *Setosphaeria sp* SCSIO41009.2 [35]
PPARγ	butyrolactone I	marine fungus *A. terreus* [36]
PPARγ	chrysogenester	jellyfish-derived Fungus *Penicillium chrysogenum* J08NF-4 [37]
PPARγ	gracilioether B plakilactone C	marine sponge *Plakinastrella mamillaris* [38]
PPARγ	paecilocin A	jellyfish-derived fungus *Paecilomyces variotii* [39]
PPARγ	psammaplin A	marine sponge *Pseudoceratina rhax* (Aplysinelliae) [40]
PPARγ	furanylidene acetates	marine sponge *Plakortis simplex* [41]
PPARγ	plakdiepoxide	marine sponge *Plakortis simplex* [41]
PPARδ	phosphoiodyns A and B	Korean sponge *Placospongia sp.* [42]
PPARα/γ	astaxanthin	Seafood [43]
PPARα/γ	C16 Oxo-Fatty Acids	diatom *Chaetoceros karianus* [44]
PPARα/γ	caulerpin	green alga *Caulerpa cylindracea* [45]
PPARα/γ	muqubilin	sponge *Diacarnus cf. spinopoculum* [46]
PPARα/γ	sargaquinoic and sargahydroquinoic acids	brown alga *Sargassum yezoense* [47]
PPARα/γ	monotriajaponide	marine sponge *Plakortis simplex* [41]
PPARα/δ	2,4-dimethyl-4-hydroxy-16-phenylhexadecanoic acid 1,4-lactone	deep-water sponge *Plakortis nigra* [48]
PPARα/γ/δ	PUFA	red alga *Chondrus crispus/* [49] brown alga *Lessonia spicata* [50]
